# Association between potassium supplementation and the occurrence of acute kidney injury in patients with hypokalemia administered liposomal amphotericin B: a nationwide observational study

**DOI:** 10.1186/s12882-021-02450-7

**Published:** 2021-06-30

**Authors:** Yuki Ota, Yoko Obata, Takahiro Takazono, Masato Tashiro, Tomotaro Wakamura, Akinori Takahashi, Yui Shiozawa, Taiga Miyazaki, Tomoya Nishino, Koichi Izumikawa

**Affiliations:** 1 Department of Nephrology, Nagasaki University Hospital, 1-7-1 Sakamoto, Nagasaki, 852-8501 Japan; 2 Department of Infectious Diseases, Nagasaki University Graduate School of Biomedical Sciences, 1-7-1 Sakamoto, Nagasaki, 852-8501 Japan; 3 Department of Respiratory Medicine, Nagasaki University Hospital, 1-7-1 Sakamoto, Nagasaki, 852-8501 Japan; 4 Nagasaki University Infection Control and Education Center, Nagasaki University Hospital, 1-7-1 Sakamoto, Nagasaki, 852-8501 Japan; 5 Medical Affairs Division, Sumitomo Dainippon Pharma Co., Ltd, 1-13-1 Kyobashi, Chuo-ku, Tokyo, 104-8356 Japan; 6 Deloitte Tohmatsu Consulting LLC, Marunouchi Nijubashi Building, 3-2-3 Marunouchi, Chiyoda-ku, Tokyo, 100-8361 Japan

**Keywords:** Liposomal amphotericin B, Hypokalemia, Potassium supplementation, Acute kidney injury, Observational study

## Abstract

**Background:**

Hypokalemia and acute kidney injury (AKI) occur in patients administered liposomal amphotericin B (L-AMB), a wide-spectrum anti-fungicidal drug. However, the association between potassium supplementation and the occurrence of AKI in patients with hypokalemia who were administered L-AMB is not well understood.

**Methods:**

Using nationwide claims data and laboratory data, the occurrence of AKI during L-AMB treatment was retrospectively compared between patients with hypokalemia who were or were not supplemented with potassium and between those adequately or inadequately supplemented with potassium (serum potassium levels corrected to ≥3.5 mEq/L or remained < 3.5 mEq/L, respectively) before or after L-AMB treatment initiation.

**Results:**

We identified 118 patients who developed hypokalemia before L-AMB treatment initiation (43 received potassium supplementation [25 adequate and 18 inadequate supplementation] and 75 did not receive potassium supplementation), and 117 patients who developed hypokalemia after L-AMB initiation (79 received potassium supplementation [including 23 adequate and 15 inadequate supplementation] and 38 did not receive potassium supplementation). The occurrence of any stage of AKI was similar between patients with hypokalemia, regardless of potassium supplementation (i.e., before L-AMB treatment initiation [supplementation, 51%; non-supplementation, 45%; *P* = 0.570] or after L-AMB initiation [supplementation, 28%; non-supplementation, 32%; *P* = 0.671]). After adjusting for confounding factors, we found that the occurrence of any stage of AKI was not associated with potassium supplementation before L-AMB initiation (odds ratio [OR]: 1.291, 95% confidence interval [CI]: 0.584–2.852, *P* = 0.528) or after L-AMB initiation (OR: 0.954, 95% CI: 0.400–2.275, *P* = 0.915). The occurrence of any stage of AKI tended to decline in patients with hypokalemia who were adequately supplemented with potassium (44%) before, but not after, L-AMB initiation relative to that in patients inadequately supplemented with potassium (61%), however this result was not significant (*P* = 0.358).

**Conclusion:**

Potassium supplementation was not associated with any stage of AKI in patients with hypokalemia who were administered L-AMB.

**Supplementary Information:**

The online version contains supplementary material available at 10.1186/s12882-021-02450-7.

## Background

Invasive fungal infections frequently occur in immunocompromised and critically ill patients and are associated with high morbidity and mortality [1, 2]. Amphotericin B (AMB) is a broad-spectrum anti-fungicidal drug that is used against yeasts and molds that cause mycoses, such as aspergillosis, candidiasis**,** cryptococcosis, and mucormycosis [3]. However, the high occurrence of toxicity associated with AMB, including nephrotoxicity, liver disorder, or hypokalemia, has limited its use [3, 4]. Liposomal amphotericin B (L-AMB), which contains AMB encapsulated in a lipid membrane, was developed to reduce AMB toxicity, without reducing its antifungal activity [3, 5]. However, regardless of its reduced nephrotoxicity, physicians are reluctant to prescribe L-AMB owing to its association with the occurrence of renal dysfunction or hypokalemia [6].

L-AMB may induce nephrotoxicity through tubular injury or renal vasoconstriction [7, 8]. Tubular injury may be induced by intramembranous pore formation or vacuolation of the epithelial cells in the distal convoluted tubule [9], while vasoconstriction may be induced by direct vasoconstrictor effects that might be initiated by the depolarization-induced opening of calcium channels [10]. This tubular injury may, in part, be responsible for L-AMB-induced hypokalemia [9]. Tubular injury may lead to increased permeability of the distal convoluted tubule and a subsequent increase in urinary potassium secretion through tubular Na^+^, K^+^-ATPase [9]. In addition, tubular injury may result in a defective the distal tubule H^+^, K^+^-ATPase, or renal tubular acidosis, causing increased potassium elimination [9].

Hypokalemia, especially if persistent, is associated with renal dysfunction, such as degeneration of the convoluted tubules [11]. This degeneration may be caused by tubular cytoplasmic vacuolization, cyst formation and interstitial fibrosis [12, 13]. Additionally, short-term hypokalemia developed before L-AMB initiation is an independent risk factor for severe acute kidney injury (AKI) stage 2 or 3 during L-AMB treatment [14]. Thus, intervention for hypokalemia in patients administered L-AMB could be essential to prevent AKI progression.

Several studies conducted in single facilities showed that potassium supplementation helps prevent hypokalemia during L-AMB treatment [15, 16]. However, association between potassium supplementation and the occurrence of AKI in patients with hypokalemia administered L-AMB has not been well understood. Using nationwide claims data for Japan and laboratory data, we aimed to evaluate the association between potassium supplementation and the occurrence of AKI development during L-AMB treatment in patients with hypokalemia, which developed before or after L-AMB treatment initiation. We compared AKI occurrence during L-AMB treatment between patients with hypokalemia who were or were not supplemented with potassium as well as between patients who were adequately or inadequately supplemented with potassium before or after treatment initiation.

## Methods

### Data source

This retrospective, multicenter, observational study was based on data retrieved between April 2008 and January 2018 from an electronic medical information database (Medical Data Vision Co., Ltd.) [14]. This database contains diagnosis procedure combination hospital data, medical fee reimbursement claims, and clinical laboratory test data from 345 facilities in Japan. The database included information regarding age, sex, diagnosis, and comorbidities at admission, coded using the International Classification of Diseases, 10th Revision codes. The database also contained dosages and administration dates of drugs as well as interventional procedures during hospitalization. Subjects were admitted to public, private, or government hospitals, but not university hospitals or facilities that had less than 200 beds.

### Study design

As described by Takazono et al. [14], we identified 507 subjects administered L-AMB during hospitalization. Thereafter, two study populations were independently selected. First, patients who developed hypokalemia before L-AMB treatment initiation were identified as subjects with serum potassium levels < 3.5 mEq/L between 7 and 2 days before L-AMB initiation. Among these, subjects supplemented with potassium were defined as patients treated with potassium L-aspartate, potassium gluconate, or potassium chloride between the day after hypokalemia onset and the day before the most recent date of potassium measurement until L-AMB initiation. Subjects supplemented with potassium were divided into those with adequate supplementation defined as a correction of the serum potassium levels to ≥3.5 mEq/L on the most recent date of potassium measurement until L-AMB initiation and those with inadequate supplementation defined as incomplete serum potassium correction (i.e., < 3.5 mEq/L). Second, patients who developed hypokalemia after L-AMB initiation were identified as subjects who met the following criteria: 1) serum potassium levels ≥3.5 mEq/L on the most recent date of potassium measurement until L-AMB initiation, and 2) serum potassium levels < 3.5 mEq/L between the day after L-AMB initiation and 2 days before the onset of AKI or the day before L-AMB termination. Among these, subjects supplemented with potassium were defined as patients treated with potassium between the day after hypokalemia onset and the day before AKI onset or the day of L-AMB termination. Further, to evaluate AKI, we selected patients who had either serum creatinine levels measured or underwent dialysis between the day after potassium supplementation initiation (supplementation group) or 2 days after hypokalemia onset (non-supplementation group) and 7 days after L-AMB termination. To assess the association between the adequacy of potassium supplementation and AKI, we selected patients who had received potassium supplementation and had their serum potassium levels measured between the day after supplementation termination and the day before AKI onset or 6 days after L-AMB termination. Adequate potassium supplementation was defined as the correction of serum potassium levels to ≥3.5 mEq/L on the day of serum potassium measurement immediately after the day of potassium supplementation termination, while inadequate potassium supplementation was defined as incomplete serum potassium correction (< 3.5 mEq/L). Further, to evaluate AKI, we assessed patients who had either serum creatinine levels measured or underwent dialysis between the day after the day of adequacy judgement was made (i.e. adequate or inadequate supplementation with potassium) and 7 days after L-AMB termination.

### Assessments

AKI and estimated glomerular filtration rate (eGFR) were defined as described by Takazono et al. [14]. Briefly, based on the KDIGO AKI criteria, AKI was defined as a ≥ 1.5-fold increase within 7 days or ≥ 0.3 mg/dL increase within 2 days in serum creatinine (Cr) levels. AKI patients were assigned to either of three stages: stage 1, ≥1.5- to < 2-fold increase or ≥ 0.3 mg/dL increase in Cr; stage 2, ≥2- to < 3-fold increase in Cr; stage 3, ≥3-fold increase in Cr, ≥4.0 mg/dL of Cr or dialysis initiation. eGFR was calculated with the following formula for Japanese individuals:

$$ \mathrm{eGFR}\ \left(\mathrm{mL}/\min \right)=\left[194\times \mathrm{Cr}\ \mathrm{concentration}\ {\left(\mathrm{mg}/\mathrm{dL}\right)}^{-1.094}\times \mathrm{age}\ {\left(\mathrm{years}\right)}^{-0.287}\left(\times 0.739\ \mathrm{for}\ \mathrm{women}\right)/1.73\ {\mathrm{m}}^2\right]\times \mathrm{Body}\ \mathrm{surface}\ \mathrm{area}\ \left({\mathrm{m}}^2\right) $$

$$ \mathrm{Body}\ \mathrm{surface}\ \mathrm{area}\ \left({\mathrm{m}}^2\right)=0.007184\times {\left[\mathrm{weight}\ \left(\mathrm{kg}\right)\right]}^{0.425}\times {\left[\mathrm{height}\ \left(\mathrm{cm}\right)\right]}^{0.725} $$

To calculate eGFR at baseline, the minimum level of Cr measured between 180 and 7 days before L-AMB initiation was used.

Sex and age were obtained on the first day of the month of L-AMB initiation. Comorbidities and fungal infections were identified using the corresponding ICD-10 codes which were registered on the month of L-AMB initiation. L-AMB treatment duration was defined as the time from treatment initiation to discontinuation (interval ≥ 8 days). The treatment department was defined as that which initiated L-AMB treatment.

Variables associated with serum potassium levels and potassium supplementation were assessed at different periods between patients with hypokalemia before and after L-AMB initiation. The duration of hypokalemia was defined as the consecutive duration of having a serum potassium level < 3.5 mEq/L between 7 days before and the day of L-AMB initiation for patients with hypokalemia before L-AMB initiation, while that was also defined as the consecutive duration of having a serum potassium level < 3.5 mEq/L between the day after L-AMB initiation and the day before AKI or 7 days after L-AMB termination for patients with hypokalemia after L-AMB initiation. For patients with hypokalemia before L-AMB initiation, the minimum level of serum potassium was evaluated between 7 days before and the day of L-AMB initiation for patients who were or were not supplemented with potassium or between 7 days before L-AMB initiation and the day before supplementation initiation for patients who were adequately or inadequately supplemented with potassium. For patients with hypokalemia after L-AMB initiation, the minimum serum potassium level was also evaluated between the day after L-AMB initiation and the day before AKI or 7 days after L-AMB termination for patients who were or were not supplemented with potassium or between the day after L-AMB initiation and the day before potassium supplementation initiation for patients who were adequately or inadequately supplemented with potassium. The average serum potassium levels and the duration of potassium supplementation following hypokalemia onset, defined as the time from supplementation initiation to discontinuation (interval ≥ 2 days), were evaluated between 7 days before and the day of L-AMB initiation for patients with hypokalemia before L-AMB initiation, or between the day after L-AMB initiation and the day before AKI or 7 days after L-AMB termination for patients with hypokalemia after L-AMB initiation. Daily and cumulative potassium dosing during potassium supplementation duration were also calculated.

We identified drug treatments during the different periods based on the types of drugs or subjects. Pretreatment with potassium-related drugs was identified between 7 days before and the day of L-AMB initiation. However, only for patients who were adequately or inadequately supplemented with potassium before L-AMB initiation, pretreatment with potassium-related drugs was identified between 7 days before L-AMB initiation and the most recent potassium measurement until L-AMB initiation. Concomitant treatment with potassium-related drugs was identified between the day after L-AMB initiation and the day before AKI or 7 days after L-AMB termination. However, only for patients who were adequately or inadequately supplemented with potassium after L-AMB initiation, concomitant treatment with potassium-related drugs was identified between the day after L-AMB initiation and the day of serum potassium measurement immediately following the day of potassium supplementation termination. Pretreatment with AKI-related drugs was identified between the admission date and the day before L-AMB initiation, while concomitant treatment with AKI-related drugs was identified between the day of L-AMB initiation and termination.

### Statistical analysis

The occurrence of AKI (any or each stage) during L-AMB treatment was compared between patients with hypokalemia who were or were not supplemented with potassium or between those adequately or inadequately supplemented with potassium before or after L-AMB initiation using the Fisher’s exact test. To exclude the effect of the confounding factors on renal dysfunction, logistic regression analysis was conducted using the occurrence of AKI (any stage, stage 2 or 3, or stage 2 or 3 excluding dialysis) as the dependent variable. We have selected 13 independent variables associated with AKI in patients who developed hypokalemia and were administered L-AMB. These variables included those related to potassium supplementation, age, sex, comorbidities (diabetes mellitus, hypertension, heart failure), severe infection (defined as treatment with catecholamine), baseline eGFR, L-AMB cumulative dosing, minimum serum potassium levels, and treatment with AKI-related drugs (angiotensin-converting enzyme inhibitor/angiotensin receptor blocker [ACE inhibitors/ARB], loop/thiazide diuretic drugs, or immunosuppressant/steroid). For logistic regression analysis, minimum serum potassium and treatment with drugs were evaluated between 7 days before L-AMB initiation and the day before AKI or 7 days after L-AMB termination. The variables were subjected to univariate binomial logistic regression analysis. Potassium supplementation and variables with a *P*-value of < 0.2 in univariate logistic regression analysis were subjected to multivariate logistic regression analysis. Continuous variables (L-AMB cumulative dosing or minimum serum potassium levels) were divided into two groups using the cut-off value calculated with receiver operating characteristic curves when analyzed with the multivariate logistic regression model. The association between adequate potassium supplementation and sex was evaluated using univariate logistic regression analysis with adequate or inadequate potassium supplementation as the dependent variable and with sex as the independent variable in patients with hypokalemia who were supplemented with potassium before L-AMB initiation. The association between drug treatments and AKI was evaluated using univariate logistic regression analysis with any stage of AKI as the dependent variable and drug treatments as the independent variable in patients with hypokalemia who were or were not supplemented with potassium before L-AMB initiation. Odds ratio (OR), 95% confidence interval (CI) and variance inflation factor (VIF) were calculated. To evaluate patient characteristics, the Welch’s *t-*test was used to compare the two groups for continuous variables, while the Fisher’s exact test was used for two categorical variables.

## Results

### Study population

**Fig. 1:**
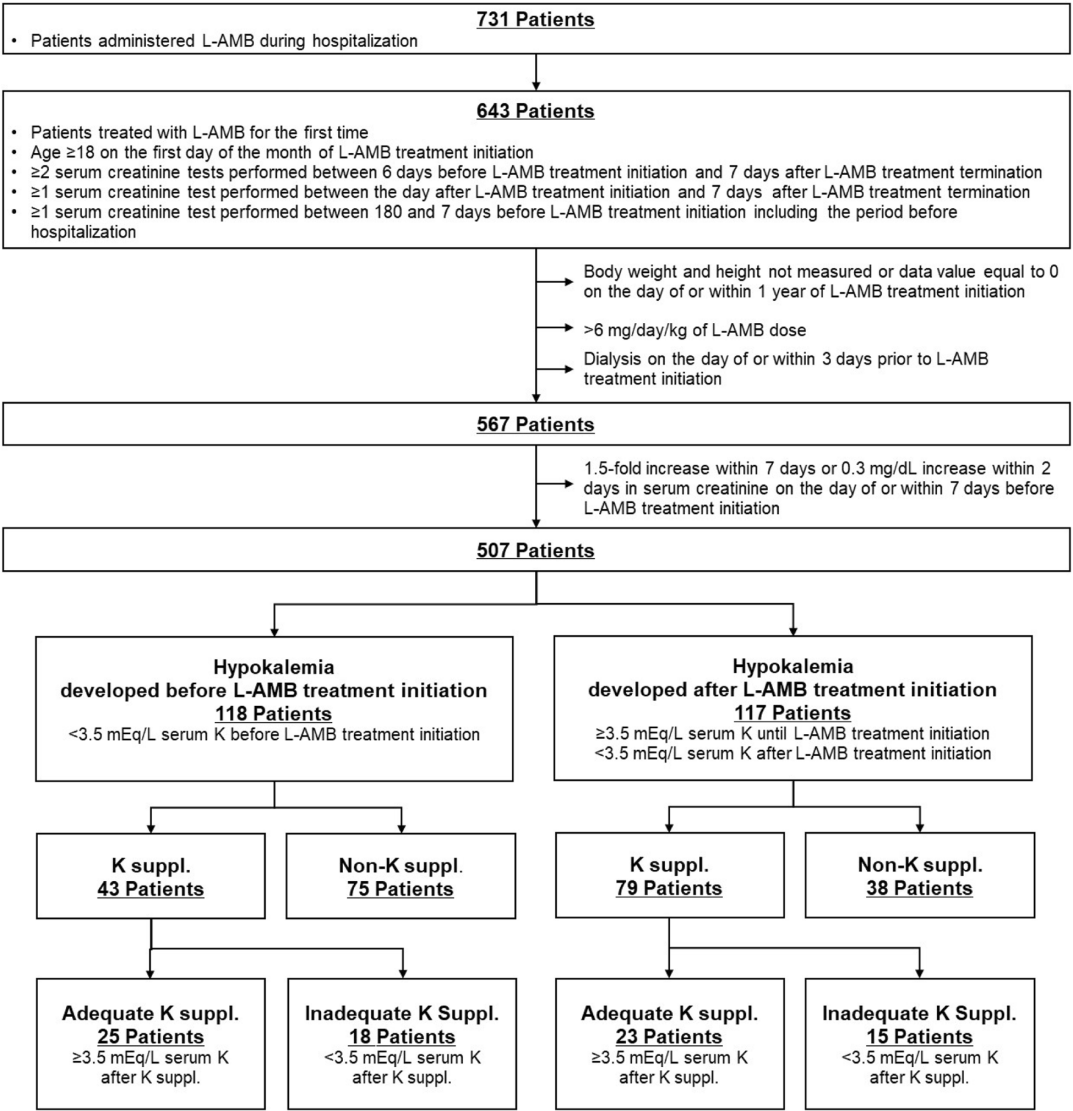
Flow chart for patient selection. K: potassium; L-AMB: liposomal-amphotericin B; suppl.: supplementation

As shown in Fig. 1, by applying the criteria and definitions described in the Materials and Methods, we identified 118 patients who developed hypokalemia before L-AMB treatment initiation (43 received potassium supplementation and 75 did not receive potassium supplementation). Of the 43 patients who were supplemented with potassium, 25 received adequate supplementation and 18 received inadequate supplementation. Additionally, 117 patients who developed hypokalemia after L-AMB initiation were identified: 79 received potassium supplementation and 38 did not receive potassium supplementation. Of the 79 patients, 23 received adequate supplementation with potassium while 15 received inadequate supplementation with potassium.

### Association between potassium supplementation and AKI occurrence in patients with hypokalemia and administered L-AMB

The characteristics of patients with hypokalemia who were or were not supplemented with potassium before or after L-AMB treatment are presented in Table 1. In patients who developed hypokalemia before L-AMB treatment initiation, the minimum serum potassium levels and potassium levels upon hypokalemia onset were lower in patients supplemented with potassium before L-AMB initiation than in patients who did not receive potassium supplementation. The proportion of patients treated with potassium-sparing diuretic agents until L-AMB initiation was higher in patients supplemented with potassium than in patients who did not receive potassium supplementation. The occurrence of any stage of AKI was comparable between both populations (supplementation 22/43, 51%; non-supplementation 34/75, 45%; *P* = 0.570) (Table 2). To exclude the effect of the confounding factors on renal dysfunction, we conducted logistic regression analysis. Through univariate logistic regression analysis, four variables were associated with any stage of AKI (*P* < 0.2) (< 65 years of age, hypertension, higher minimum serum potassium level, and treatment with ACE inhibitors/ARB) (Table 3). Using these variables and potassium supplementation in multivariate regression analysis, we found that potassium supplementation before L-AMB initiation was not associated with any stage of AKI (OR: 1.291, 95% CI: 0.584–2.852, *P* = 0.528) (Table 3). Moreover, potassium supplementation before L-AMB initiation was associated with neither AKI stage 2 or 3 (OR: 0.902, 95% CI: 0.364–2.237, *P* = 0.824) (Table S1) nor AKI stage 2 or 3 excluding dialysis (OR: 0.710, 95% CI: 0.274–1.845, *P* = 0.482) (Table S2).

**Table 1:** Characteristics of hypokalemic patients with or without potassium supplementation administered L-AMB

	Patients who developed hypokalemia before L-AMB treatment initiation				Patients who developed hypokalemia after L-AMB treatment initiation			
Patient characteristics	Overall (***N*** = 118)	K suppl. (***N*** = 43)	Non-K suppl. (***N*** = 75)	***P***-value	Overall (***N*** = 117)	K suppl. (***N*** = 79)	Non-K suppl. (***N*** = 38)	***P***-value
Sex, male	71 (60%)	27 (63%)	44 (59%)	0.700	70 (60%)	49 (62%)	21 (55%)	0.548
Age, years	66.8 ± 14.3	66.9 ± 14.7	66.7 ± 14.0	0.953	66.7 ± 14.3	66.6 ± 14.5	67.0 ± 13.8	0.889
Comorbidities								
Diabetes mellitus	43 (36%)	14 (33%)	29 (39%)	0.555	36 (31%)	26 (33%)	10 (26%)	0.526
Hypertension	41 (35%)	15 (35%)	26 (35%)	1.000	44 (38%)	33 (42%)	11 (29%)	0.223
Heart failure	28 (24%)	11 (26%)	17 (23%)	0.823	18 (15%)	**18 (23%)**	**0 (0%)**	**< 0.001**
Baseline eGFR (mL/min)	100.8 ± 48.2	111.9 ± 57.1	94.5 ± 41.0	0.087	98.1 ± 50.0	101.4 ± 56.4	91.2 ± 31.6	0.219
L-AMB treatment								
Duration (days)	15.1 ± 13.3	16.3 ± 15.8	14.4 ± 11.7	0.493	21.2 ± 15.9	**24.7 ± 17.6**	**13.8 ± 7.3**	**< 0.001**
Daily dosing (mg/kg/day)	2.4 ± 0.9	2.5 ± 0.7	2.4 ± 0.9	0.847	2.6 ± 0.8	**2.8 ± 0.9**	**2.4 ± 0.6**	**0.019**
Cumulative dosing (mg/kg)	37.2 ± 35.8	41.6 ± 41.7	34.7 ± 31.6	0.350	57.6 ± 49.9	**68.9 ± 55.6**	**34.2 ± 20.7**	**< 0.001**
Serum K								
Duration of hypokalemia (days)	3.8 ± 2.4	4.1 ± 2.5	3.6 ± 2.4	0.325	6.7 ± 7.0	7.2 ± 8.0	5.8 ± 4.3	0.222
Minimum K (mEq/L)	3.0 ± 0.3	**2.9 ± 0.3**	**3.0 ± 0.3**	**0.009**	2.8 ± 0.4	**2.7 ± 0.4**	**2.9 ± 0.3**	**0.020**
K level of hypokalemia onset (mEq/L)	3.1 ± 0.3	**3.1 ± 0.2**	**3.2 ± 0.2**	**0.017**	3.1 ± 0.3	3.1 ± 0.3	3.2 ± 0.2	0.068
Average K (mEq/L)	3.4 ± 0.4	3.3 ± 0.3	3.4 ± 0.4	0.265	3.4 ± 0.4	3.4 ± 0.5	3.3 ± 0.3	0.141
K suppl.								
Duration of supplementation (days)	NA	3.9 ± 2.2	NA	NA	NA	9.8 ± 11.5	NA	NA
Duration from hypokalemia onset to K suppl. (days)	NA	1.6 ± 1.1	NA	NA	NA	2.5 ± 2.9	NA	NA
Daily dosing (mEq/day)	NA	38.6 ± 22.7	NA	NA	NA	43.2 ± 28.8	NA	NA
Cumulative dosing (mEq)	NA	142.3 ± 110.1	NA	NA	NA	393.7 ± 541.7	NA	NA
Fungal infection								
Aspergillosis	29 (25%)	15 (35%)	14 (19%)	0.074	37 (32%)	29 (37%)	8 (21%)	0.095
Candidiasis	9 (8%)	5 (12%)	4 (5%)	0.283	10 (9%)	7 (9%)	3 (8%)	1.000
Cryptococcosis	2 (2%)	0 (0%)	2 (3%)	0.533	4 (3%)	3 (4%)	1 (3%)	1.000
Zygomycosis	1 (1%)	1 (2%)	0 (0%)	0.364	1 (1%)	1 (1%)	0 (0%)	1.000
Aspergillosis, Candidiasis	0 (0%)	0 (0%)	0 (0%)	1.000	1 (1%)	0 (0%)	1 (3%)	0.325
Aspergillosis, Candidiasis, Cryptococcosis	1 (1%)	0 (0%)	1 (1%)	1.000	0 (0%)	0 (0%)	0 (0%)	1.000
Neutropenia	5 (4%)	0 (0%)	5 (7%)	0.157	7 (6%)	5 (6%)	2 (5%)	1.000
Others	38 (32%)	15 (35%)	23 (31%)	0.685	32 (27%)	22 (28%)	10 (26%)	1.000
Unknown	33 (28%)	**7 (16%)**	**26 (35%)**	**0.035**	25 (21%)	**12 (15%)**	**13 (34%)**	**0.029**
Treatment department								
Hematology	78 (66%)	31 (72%)	47 (63%)	0.320	79 (68%)	57 (72%)	22 (58%)	0.143
The internal department except for hematology	34 (29%)	9 (21%)	25 (33%)	0.205	28 (24%)	15 (19%)	13 (34%)	0.104
The surgical department	5 (4%)	2 (5%)	3 (4%)	1.000	9 (8%)	6 (8%)	3 (8%)	1.000
Others	1 (1%)	1 (2%)	0 (0%)	0.364	1 (1%)	1 (1%)	0 (0%)	1.000
Pretreatment potassium-related drugs								
Insulin	46 (39%)	21 (49%)	25 (33%)	0.118	32 (27%)	22 (28%)	10 (26%)	1.000
ACE inhibitors, ARB	10 (8%)	2 (5%)	8 (11%)	0.323	10 (9%)	7 (9%)	3 (8%)	1.000
Sodium bicarbonate	14 (12%)	6 (14%)	8 (11%)	0.768	12 (10%)	7 (9%)	5 (13%)	0.522
Potassium citrate	0 (0%)	0 (0%)	0 (0%)	1.000	0 (0%)	0 (0%)	0 (0%)	1.000
Diuretic drugs								
Loop	65 (55%)	24 (56%)	41 (55%)	1.000	42 (36%)	31 (39%)	11 (29%)	0.310
Thiazide	4 (3%)	1 (2%)	3 (4%)	1.000	2 (2%)	2 (3%)	0 (0%)	1.000
Potassium-sparing	13 (11%)	**9 (21%)**	**4 (5%)**	**0.014**	3 (3%)	3 (4%)	0 (0%)	0.550
β-agonist	13 (11%)	5 (12%)	8 (11%)	1.000	8 (7%)	6 (8%)	2 (5%)	1.000
β-blocker	9 (8%)	3 (7%)	6 (8%)	1.000	2 (2%)	1 (1%)	1 (3%)	0.546
Concomitant potassium-related drugs								
Insulin	41 (35%)	15 (35%)	26 (35%)	1.000	43 (37%)	30 (38%)	13 (34%)	0.838
ACE inhibitors, ARB	7 (6%)	3 (7%)	4 (5%)	0.704	12 (10%)	10 (13%)	2 (5%)	0.332
Sodium bicarbonate	16 (14%)	5 (12%)	11 (15%)	0.783	6 (5%)	5 (6%)	1 (3%)	0.662
Potassium citrate	0 (0%)	0 (0%)	0 (0%)	1.000	0 (0%)	0 (0%)	0 (0%)	1.000
Diuretic drugs								
Loop	63 (53%)	24 (56%)	39 (52%)	0.706	58 (50%)	43 (54%)	15 (39%)	0.167
Thiazide	3 (3%)	0 (0%)	3 (4%)	0.299	2 (2%)	2 (3%)	0 (0%)	1.000
Potassium-sparing	12 (10%)	5 (12%)	7 (9%)	0.756	15 (13%)	**15 (19%)**	**0 (0%)**	**0.002**
β-agonist	9 (8%)	3 (7%)	6 (8%)	1.000	13 (11%)	9 (11%)	4 (11%)	1.000
β-blocker	7 (6%)	3 (7%)	4 (5%)	0.704	6 (5%)	5 (6%)	1 (3%)	0.662

Bold values indicate statistically significant *P*-values (*P* < 0.05). Other fungal infections included unclassified or unspecified mycosis. Categorical variables are presented as frequencies and proportions (%), while continuous variables are expressed as mean ± standard deviation. The Welch’s *t*-test was used to compare two groups for continuous variables, while the Fisher’s exact test was used for two categorical variables. *ACE inhibitor/ARB* angiotensin-converting enzyme inhibitor/angiotensin receptor blocker, *eGFR* estimated glomerular filtration rate, *L-AMB* liposomal-amphotericin B, *K* potassium, *NA* not analyzed, *suppl* supplementation.

**Table 2:** AKI occurrence in hypokalemic patients with or without potassium supplementation administered L-AMB

	Patients who developed hypokalemia before L-AMB treatment initiation				Patients who developed hypokalemia after L-AMB treatment initiation			
	Overall (***N*** = 118)	K suppl. (***N*** = 43)	Non-K suppl. (***N*** = 75)	***P***-value	Overall (***N*** = 117)	K suppl. (***N*** = 79)	Non-K suppl. (***N*** = 38)	***P***-value
AKI (any stage)	56 (47%)	22 (51%)	34 (45%)	0.570	34 (29%)	22 (28%)	12 (32%)	0.671
Stage 1	25 (21%)	11 (26%)	14 (19%)	0.483	18 (15%)	12 (15%)	6 (16%)	1.000
Stage 2 or 3	31 (26%)	11 (26%)	20 (27%)	1.000	16 (14%)	10 (13%)	6 (16%)	0.775
Stage 2	22 (19%)	6 (14%)	16 (21%)	0.462	8 (7%)	6 (8%)	2 (5%)	1.000
Stage 3	9 (8%)	5 (12%)	4 (5%)	0.283	8 (7%)	4 (5%)	4 (11%)	0.435
Dialysis	2 (2%)	2 (5%)	0 (0%)	0.131	1 (1%)	1 (1%)	0 (0%)	1.000

AKI stage 1, Cr ≥1.5 to < 2-fold or ∆Cr ≥0.3 mg/dL; stage 2, Cr ≥2 to < 3-fold; stage 3, Cr ≥3-fold or Cr ≥4.0 mg/dL or dialysis. Variables are presented as frequencies and proportions (%). *P*-values were calculated using the Fisher’s exact test. *AKI* acute kidney injury, *Cr* creatinine, *K* potassium, *L-AMB* liposomal-amphotericin B, *suppl*. supplementation

**Table 3:** Logistic regression analysis of the factors associated with AKI in hypokalemic patients before L-AMB initiation

	Univariate regression		Multivariate regression		
Variables	OR (95% CI)	***P***-value	OR (95% CI)	***P***-value	VIF
K suppl., with (vs without)	1.263 (0.596–2.677)	0.542	1.291 (0.584–2.852)	0.528	1.009
Age, ≥65 years (vs < 65 years)	0.439 (0.206–0.938)	0.034	0.479 (0.217–1.056)	0.068	1.015
Sex, male (vs female)	1.204 (0.574–2.522)	0.623			
Comorbidities, with (vs without)					
Diabetes mellitus	1.263 (0.596–2.677)	0.542			
Hypertension	1.704 (0.793–3.659)	0.172	1.397 (0.617–3.163)	0.422	1.042
Heart failure	1.667 (0.708–3.924)	0.242			
Severe infection (Catecholamine treatment, with [vs without])	1.787 (0.593–5.387)	0.302			
Baseline eGFR, ≥60 mL/min (vs < 60 mL/min)	1.962 (0.627–6.139)	0.247			
L-AMB cumulative dosing (mg/kg, continuous value)	1.001 (0.991–1.011)	0.875			
Minimum K (mEq/L)					
Univariate regression: continuous value	2.573 (0.889–7.449)	0.081	2.398 (1.038–5.537)	0.041	1.022
Multivariate regression: ≥2.91 mEq/L (vs < 2.91 mEq/L)					
Drug treatment, with (vs without)					
ACE inhibitors/ARB	2.417 (0.686–8.517)	0.170	2.063 (0.536–7.946)	0.293	1.056
Loop/thiazide diuretic drugs	0.736 (0.343–1.582)	0.433			
Immunosuppressant/Steroid	0.585 (0.206–1.660)	0.314			

Logistic regression analysis was conducted using the occurrence of any stage of AKI as the dependent variable. Thirteen independent variables related to AKI were subjected to univariate binomial logistic regression analysis. K suppl. and variables with a *P*-value of < 0.2 in univariate logistic regression analysis were subjected to multivariate logistic regression analysis. The continuous variable (i.e., minimum K level) was divided into two groups based on the cut-off value when analyzed using the multivariate logistic regression model. OR, 95% CI, and VIF were calculated. *AKI* acute kidney injury, *ACE inhibitors/ARB* angiotensin-converting enzyme inhibitor/angiotensin receptor blocker, *CI* confidence interval, *eGFR* estimated glomerular filtration rate, *K* potassium, *L-AMB* liposomal-amphotericin B, *suppl*. Supplementation, *OR* odds ratio, *VIF* variance inflation factor.

Of the patients who developed hypokalemia after L-AMB initiation, those supplemented with potassium after L-AMB initiation had a higher prevalence of heart failure than those who did not receive potassium supplementation. Further, patients supplemented with potassium received L-AMB for a longer duration (24.7 days) than those who did not receive potassium supplementation (13.8 days). The daily and cumulative administration doses of L-AMB were higher in patients supplemented with potassium (2.8 ± 0.9 mg/kg/day and 68.9 ± 55.6 mg/kg, respectively) than those in patients who did not receive potassium supplementation (2.4 ± 0.6 mg/kg/day and 34.2 ± 20.7 mg/kg, respectively). The minimum serum potassium levels were lower in patients supplemented with potassium than those in patients who did not receive potassium supplementation. The proportion of patients treated with potassium-sparing diuretic agents after L-AMB initiation was higher in patients supplemented with potassium than those who did not receive potassium supplementation. The occurrence of any stage of AKI was comparable between both populations (supplementation 22/79, 28%; non-supplementation 12/38, 32%; *P* = 0.671; Table 2). We conducted logistic regression analysis to adjust for potential confounding variables. Through univariate logistic regression analysis, treatment with loop/thiazide diuretic drugs was identified as the factor associated with any stage of AKI (*P* < 0.2) (Table 4). Using this variable and potassium supplementation in multivariate regression analysis, we found that potassium supplementation conducted after L-AMB initiation was not associated with any stage of AKI (OR: 0.954, 95% CI: 0.400–2.275, *P* = 0.915; Table 4).

**Table 4:** Logistic regression analysis of the factors associated with AKI in hypokalemic patients after L-AMB initiation

	Univariate regression		Multivariate regression		
Variables	OR (95% CI)	***P***-value	OR (95% CI)	***P***-value	VIF
K suppl., with (vs without)	0.836 (0.360–1.942)	0.677	0.954 (0.400–2.275)	0.915	1.046
Age, ≥65 years (vs < 65 years)	0.884 (0.382–2.048)	0.773			
Sex, male (vs female)	1.337 (0.584–3.058)	0.492			
Comorbidities, with (vs without)					
Diabetes mellitus	0.747 (0.307–1.817)	0.520			
Hypertension	0.869 (0.379–1.995)	0.741			
Heart failure	0.657 (0.200–2.162)	0.490			
Severe infection (Catecholamine treatment, with [vs without])	1.129 (0.360–3.534)	0.836			
Baseline eGFR, ≥60 mL/min (vs < 60 mL/min)	2.704 (0.572–12.792)	0.210			
L-AMB cumulative dosing (mg/kg, continuous value)	0.994 (0.985–1.004)	0.237			
Minimum K (mEq/L, continuous value)	1.662 (0.568–4.870)	0.354			
Drug treatment, with (vs without)					
ACE inhibitors/ARB	1.418 (0.438–4.588)	0.560			
Loop/thiazide diuretic drugs	0.548 (0.245–1.227)	0.143	0.553 (0.243–1.261)	0.159	1.046
Immunosuppressant/Steroid	0.783 (0.285–2.150)	0.634			

Logistic regression analysis was conducted using the occurrence of any stage of AKI as the dependent variable. Thirteen independent variables associated with AKI were subjected to univariate binomial logistic regression analysis. K suppl. and variable with a *P*-value of < 0.2 in univariate logistic regression analysis were subjected to multivariate logistic regression analysis. OR, 95% CI, and VIF were calculated. *AKI* acute kidney injury, *ACE inhibitors/ARB* angiotensin-converting enzyme inhibitor/angiotensin receptor blocker, *CI* confidence interval, *eGFR* estimated glomerular filtration rate, *K* potassium, *L-AMB* liposomal-amphotericin B, *suppl*. Supplementation, *OR* odds ratio, *VIF* variance inflation factor.

### Association between adequate potassium supplementation and AKI occurrence in patients with hypokalemia administered L-AMB

The characteristics of patients with hypokalemia who were adequately or inadequately supplemented with potassium before or after L-AMB treatment initiation are shown in Table S3. Of the patients who developed hypokalemia and received potassium supplementation before L-AMB initiation, patients adequately supplemented with potassium had a lower prevalence of heart failure and higher average concentrations of serum potassium than those inadequately supplemented with potassium. Patients inadequately supplemented with potassium suffered from hypokalemia for a longer period than those adequately supplemented with potassium. The occurrence of any stage of AKI was slightly though not significantly in patients adequately supplemented with potassium compared with that in patients inadequately supplemented with potassium (adequate: 11/25, 44%; inadequate: 11/18, 61%; *P* = 0.358) (Table 5). Further, after stratifying subjects according to their characteristics, we found that female adequately supplemented with potassium had a lower occurrence of AKI stage 2 or 3 than females inadequately supplemented with potassium (adequate: 0/7, 0%; inadequate: 5/9, 56%; *P* = 0.034; Table S4).

**Table 5:** AKI occurrence in hypokalemic patients adequately or inadequately supplemented with potassium administered L-AMB

	Patients who developed hypokalemia before L-AMB treatment initiation			Patients who developed hypokalemia after L-AMB treatment initiation		
	Adequate K suppl. (***N*** = 25)	Inadequate K suppl. (***N*** = 18)	***P***-value	Adequate K suppl. (***N*** = 23)	Inadequate K suppl. (***N*** = 15)	***P***-value
AKI (any stage)	11 (44%)	11 (61%)	0.358	4 (17%)	2 (13%)	1.000
Stage 1	6 (24%)	5 (28%)	1.000	2 (9%)	1 (7%)	1.000
Stage 2 or 3	5 (20%)	6 (33%)	0.480	2 (9%)	1 (7%)	1.000
Stage 2	3 (12%)	3 (17%)	0.683	1 (4%)	1 (7%)	1.000
Stage 3	2 (8%)	3 (17%)	0.634	1 (4%)	0 (0%)	1.000
Dialysis	1 (4%)	1 (6%)	1.000	0 (0%)	0 (0%)	1.000

Adequate potassium supplementation was defined as the correction of serum potassium levels to ≥3.5 mEq/L, while inadequate potassium supplementation was defined as incomplete serum potassium correction (i.e., < 3.5 mEq/L). AKI stage 1, Cr ≥1.5 to < 2-fold or ∆Cr ≥0.3 mg/dL; stage 2, Cr ≥2 to < 3-fold; stage 3, Cr ≥3-fold or Cr ≥4.0 mg/dL or dialysis. Variables are presented as frequencies and proportions (%). *P*-values were calculated using the Fisher’s exact test. *AKI* acute kidney injury, *Cr* creatinine, *K* potassium, *L-AMB* liposomal-amphotericin B, *suppl*. supplementation.

Table S3 shows that among patients who developed hypokalemia and received potassium supplementation after L-AMB initiation, those adequately supplemented with potassium were older and had lower eGFR than those inadequately supplemented with potassium. Patients inadequately supplemented with potassium suffered from hypokalemia for a longer period than those adequately supplemented with potassium. The average concentration of serum potassium was higher in patients adequately supplemented with potassium than that in patients inadequately supplemented with potassium. Further, adequately supplemented patients received potassium for a longer duration and a higher cumulative potassium dosing than the inadequately supplemented patients. The occurrence of any stage of AKI was comparable between both populations (adequate: 4/23, 17%; inadequate: 2/15, 13%; *P* = 1.000; Table 5).

## Discussion

Herein, potassium supplementation was not associated with the occurrence of any stage of AKI in patients with hypokalemia and treated with L-AMB. However, we found that patients with hypokalemia receiving adequate potassium supplementation before L-AMB initiation had a slightly lower occurrence of AKI compared with those receiving inadequate potassium supplementation, although this difference did not reach statistical significance. Although the number of subjects included in this study was limited, this tendency might be observed in patients with severe AKI (stage 2 or 3; adequate 20% vs inadequate 33%, *P* = 0.480). Additionally, the occurrence of AKI stage 2 or 3 may be especially decreased in patients with severe hypokalemia < 3.0 mEq/L and adequately supplemented with potassium (adequate 11% vs inadequate 50%, *P* = 0.131) or in those who initiated potassium supplementation on the day immediately after hypokalemia onset and were adequately supplemented with potassium (adequate 16% vs inadequate 40%, *P* = 0.193; Table S4). A significantly lower occurrence of AKI stage 2 or 3 was observed in females adequately supplemented with potassium before L-AMB initiation (adequate 0% vs inadequate 56%, *P* = 0.034), despite adequate potassium supplementation before L-AMB initiation not being associated with sex (OR 2.571, 95% CI 0.721–9.167, *P* = 0.145). This difference, may be, in part, owing to the less frequent use of cytotoxic anti-cancer agents (adequate 2/7, 29% vs inadequate 9/9, 100%, *P* = 0.005) or fluoroquinolone (adequate 1/7, 14% vs inadequate 7/9, 78%; *P* = 0.041) in females prior to L-AMB initiation. Notably, cytotoxic anti-cancer agents and fluoroquinolone are known to be associated with AKI [17, 18].

The occurrence of any stage of AKI was not associated with potassium supplementation in patients with hypokalemia administered L-AMB, which was confirmed after adjusting for potential confounding variables. As L-AMB treatment decreases serum potassium levels, in a portion of the study subjects supplemented with potassium, insufficient correction or maintenance failure of serum potassium levels might lead to renal dysfunction. Considering that potassium supplementation prior to deterioration of hypokalemia effectively corrected serum potassium levels in patients treated with L-AMB [16], early potassium supplementation might be effective for preventing AKI. For patients treated with L-AMB, appropriate timing for potassium supplementation after hypokalemia onset, proper dosing or duration of potassium supplementation, as well as the potential effects on renal dysfunction must be further elucidated.

Before applying the criteria of patient selection for AKI evaluation, we observed that 43% (122/282) of the patients developed hypokalemia (< 3.5 mEq/L serum potassium) after L-AMB initiation, which agreed with the value reported in another study [19]. In patients who developed hypokalemia after L-AMB initiation, the time from hypokalemia onset to AKI onset tended to be longer in patients supplemented with potassium than in those who did not receive potassium supplementation (supplementation: 11.0 ± 10.6 days, non-supplementation: 6.3 ± 3.7 days, *P* = 0.073). The time from L-AMB initiation to hypokalemia onset was similar between both populations (supplementation: 4.1 ± 2.4 days, non-supplementation: 4.6 ± 4.2 days, *P* = 0.724). Potassium supplementation was promptly performed within 1.5 ± 1.3 days after hypokalemia onset. Thus, potassium supplementation might be effective at delaying AKI development in patients who develop hypokalemia after L-AMB initiation.

Among patients who developed hypokalemia after L-AMB initiation, those supplemented with potassium had a longer duration of L-AMB treatment than those who did not receive potassium supplementation, thereby aligning with a prior finding [16]. Evidently, such findings were observed in patients who did not develop AKI (supplementation: 27.3 ± 18.3 days, non-supplementation: 12.3 ± 6.3 days, *P* < 0.001). However, in patients who developed AKI, no remarkable difference was observed (supplementation, 18.0 ± 13.2 days; non-supplementation, 16.9 ± 8.2 days, *P* = 0.777). Patients requiring long-term treatment with L-AMB might often be supplemented with potassium. Moreover, preventing AKI might be important to continue L-AMB treatment.

Hypokalemia that persists for at least 1 month could be associated with vacuolar lesions in the epithelial cells of the proximal or distal tubule [20]. Moreover, prolonged hypokalemia could be a risk factor for interstitial nephritis or fibrosis, tubular atrophy, and cyst formation, thereby causing severe renal dysfunction [20]. In patients not prescribed potassium-wasting diuretics, a serum potassium level of < 3.5 mEq/L (mmol/L) was significantly associated with chronic kidney disease or end-stage renal disease [21]. Therefore, < 3.5 mEq/L of hypokalemia that persists for more than 1 month might affect renal function.

In this study, the occurrence of any stage or stage 2 or 3 of AKI was slightly though not significantly in patients aged ≥65 years who developed hypokalemia before L-AMB initiation compared with that in those aged < 65 years (Table 3; S1; S2). This might be due to the less frequent use of AKI-associated drugs in patients aged ≥65 years with hypokalemia before L-AMB initiation than in those aged < 65 years. Those drug treatments included 4th generation cephalosporins before L-AMB initiation (≥65 years: 34/73 47%, < 65 years: 32/45 71%, *P* = 0.013; association with any stage of AKI, OR: 2.216, 95% CI: 1.052–4.667, *P* = 0.036), vancomycin before L-AMB initiation (≥65 years: 26/73 36%, < 65 years: 25/45 56%, *P* = 0.038; association with any stage of AKI, OR:4.091 95% CI: 1.887–8.870, *P* < 0.001), or immunosuppressants after L-AMB initiation (≥65 years: 4/73 5%, < 65 years: 13/45 29%, *P* < 0.001; association with any stage of AKI, OR: 3.109, 95% CI: 1.020–9.481, *P* = 0.046). Thus, patients aged ≥65 years with hypokalemia before L-AMB initiation were less frequently treated with nephrotoxic drugs, which might have offered protection against the development of AKI.

This study had several limitations. First, due to its retrospective design, this study cannot prove a cause-effect relationship between the use of L-AMB and the development of hypokalemia or AKI, and is therefore merely hypothesis-generating. Second, the generalizability of the findings should be carefully considered as the database did not contain data from university hospitals that may employ infectious disease experts or facilities with fewer than 200 beds. Furthermore, tracking transfers from or to other hospitals could not be carried out. Therefore, the results might not represent the daily practice of L-AMB administration in Japan. Third, we could not evaluate AKI by assessing decreased urine volume and/or AKI biomarkers [22] as these parameters could not be obtained from the database used in this study. Lastly, due to the low number of subjects enrolled in this study, we could not evaluate the association between adequate potassium supplementation and AKI occurrence using logistic regression analysis. Therefore, further large-scale prospective studies that not only include Cr levels, but also urine volume and/or AKI biomarkers, are required to confirm the results obtained herein.

## Conclusion

In this study, potassium supplementation was not associated with the occurrence of any stage of AKI in patients with hypokalemia who were administered L-AMB.

## Supplementary Information

**Additional file 1.**

## Data Availability

The data that support the findings of this study are available from Medical Data Vision Co., Ltd. but restrictions apply to the availability of these data, which were used under license for the current study, and so are not publicly available. Data are however available from the authors upon reasonable request and with permission of Medical Data Vision Co., Ltd.

## Abbreviations

ACE inhibitor/ARB: Angiotensin-converting enzyme inhibitor/angiotensin receptor blocker
AKI: Acute kidney injury
AMB: Amphotericin B
CI: Confidence interval
Cr: Creatinine
eGFR: Estimated glomerular filtration rate
K: Potassium
L-AMB: Liposomal amphotericin B
NA: Not analyzed
OR: Odds ratio
suppl.: Supplementation
VIF: Variance inflation factor
