# Supplementary material for: Association between potassium supplementation and the occurrence of acute kidney injury in patients with hypokalemia administered liposomal amphotericin B: a nationwide observational study
Source: BMC Nephrol. 2021 Jun 30;22:240. doi: 10.1186/s12882-021-02450-7 (PMC8244132; doi:10.1186/s12882-021-02450-7)
Supplement: Supplementary file 1 — Additional file 1. [file 12882_2021_2450_MOESM1_ESM.docx]

**Table S1** Factors associated with AKI stage 2 or 3 in hypokalemic patients before L-AMB initiation.

|  | **Univariate regression** | |  | **Multivariate regression** | |  |
| --- | --- | --- | --- | --- | --- | --- |
| **Variables** | **OR (95% CI)** | **P-value** |  | **OR (95% CI)** | **P-value** | **VIF** |
| K suppl., with (vs without) | 0.945 (0.402-2.223) | 0.897 |  | 0.902 (0.364-2.237) | 0.824 | 1.000 |
| Age, ≥65 years (vs <65 years) | 0.391 (0.169-0.904) | 0.028 |  | 0.439 (0.182-1.056) | 0.066 | 1.003 |
| Sex, male (vs female) | 0.621 (0.271-1.421) | 0.259 |  |  |  |  |
| Comorbidities, with (vs without) |  |  |  |  |  |  |
| Diabetes mellitus | 0.779 (0.327-1.857) | 0.574 |  |  |  |  |
| Hypertension | 2.200 (0.949-5.099) | 0.066 |  | 2.081 (0.857-5.049) | 0.105 | 1.000 |
| Heart failure | 1.464 (0.579-3.701) | 0.420 |  |  |  |  |
| Severe infection (Catecholamine treatment, with [vs without]) | 1.024 (0.300-3.487) | 0.970 |  |  |  |  |
| Baseline eGFR, ≥60 mL/min (vs <60 mL/min) | 2.547 (0.541-11.994) | 0.237 |  |  |  |  |
| L-AMB cumulative dosing (mg/kg)  Univariate regression: continuous value  Multivariate regression: ≥23.41 mg/kg (vs <23.41 mg/kg) | 1.013 (1.002-1.024) | 0.025 |  | 3.309 (1.262-8.678) | 0.015 | 1.003 |
| Minimum K (mEq/L, continuous value) | 1.371 (0.423-4.449) | 0.599 |  |  |  |  |
| Drug treatment, with (vs without) |  |  |  |  |  |  |
| ACE inhibitors/ARB | 1.463 (0.408-5.247) | 0.559 |  |  |  |  |
| Loop/thiazide diuretic drugs | 1.353 (0.555-3.299) | 0.506 |  |  |  |  |
| Immunosuppressant/Steroid | 0.832 (0.268-2.588) | 0.751 |  |  |  |  |

Logistic regression analysis was conducted using the occurrence of AKI stage 2 or 3 (including dialysis) as the dependent variable. Thirteen independent variables associated with AKI were subjected to univariate binomial logistic regression analysis. K suppl. and variables with a P-value of <0.2 in univariate logistic regression analysis were subjected to multivariate logistic regression analysis. The continuous variable (i.e., L-AMB cumulative dosing) was divided into two groups using the cut-off value when analyzed using the multivariate logistic regression model. OR, 95% CI, and VIF were calculated. AKI: acute kidney injury; ACE inhibitors/ARB: angiotensin-converting enzyme inhibitor/angiotensin receptor blocker; CI: confidence interval; eGFR: estimated glomerular filtration rate; K: potassium; L-AMB: liposomal-amphotericin B; suppl.: supplementation; OR: odds ratio; VIF: variance inflation factor

**Table S2** Factors associated with AKI stage 2/3 excluding dialysis in hypokalemic patients before L-AMB initiation.

|  | **Univariate regression** | |  | **Multivariate regression** | |  |
| --- | --- | --- | --- | --- | --- | --- |
| **Variables** | **OR (95% CI)** | **P-value** |  | **OR (95% CI)** | **P-value** | **VIF** |
| K suppl., with (vs without) | 0.728 (0.297-1.782) | 0.487 |  | 0.710 (0.274-1.845) | 0.482 | 1.005 |
| Age, ≥65 years (vs <65 years) | 0.393 (0.167-0.924) | 0.032 |  | 0.431 (0.175-1.065) | 0.068 | 1.008 |
| Sex, male (vs female) | 0.524 (0.225-1.223) | 0.135 |  | 0.490 (0.196-1.227) | 0.128 | 1.038 |
| Comorbidities, with (vs without) |  |  |  |  |  |  |
| Diabetes mellitus | 0.728 (0.297-1.782) | 0.487 |  |  |  |  |
| Hypertension | 2.143 (0.910-5.050) | 0.081 |  | 2.249 (0.891-5.673) | 0.086 | 1.035 |
| Heart failure | 1.314 (0.506-3.413) | 0.575 |  |  |  |  |
| Severe infection (Catecholamine treatment, with [vs without]) | 0.740 (0.194-2.830) | 0.660 |  |  |  |  |
| Baseline eGFR, ≥60 mL/min (vs <60 mL/min) | 2.309 (0.489-10.903) | 0.291 |  |  |  |  |
| L-AMB cumulative dosing (mg/kg)  Univariate regression: continuous value  Multivariate regression: ≥23.41 mg/kg (vs <23.41 mg/kg) | 1.011 (1.000-1.023) | 0.042 |  | 2.740 (1.027-7.306) | 0.044 | 1.008 |
| Minimum K (mEq/L, continuous value) | 1.651 (0.491-5.553) | 0.418 |  |  |  |  |
| Drug treatment, with (vs without) |  |  |  |  |  |  |
| ACE inhibitors/ARB | 1.620 (0.450-5.834) | 0.461 |  |  |  |  |
| Loop/thiazide diuretic drugs | 1.188 (0.483-2.920) | 0.708 |  |  |  |  |
| Immunosuppressant/Steroid | 0.748 (0.239-2.338) | 0.618 |  |  |  |  |

Logistic regression analysis was conducted using the occurrence of AKI stage 2 or 3 excluding dialysis as the dependent variable. Thirteen independent variables associated with AKI were subjected to univariate binomial logistic regression analysis. K suppl. and variables with a P-value of <0.2 in univariate logistic regression analysis were subjected to multivariate logistic regression analysis. The continuous variable (i.e., L-AMB cumulative dosing) was divided into two groups using the cut-off value when analyzed using the multivariate logistic regression model. OR, 95% CI, and VIF were calculated. AKI: acute kidney injury; ACE inhibitors/ARB: angiotensin-converting enzyme inhibitor/angiotensin receptor blocker; CI: confidence interval; eGFR: estimated glomerular filtration rate; K: potassium; L-AMB: liposomal-amphotericin B; suppl.: supplementation; OR: odds ratio; VIF: variance inflation factor

**Table S3** Characteristics of hypokalemic patients adequately or inadequately supplemented with potassium administered L-AMB.

|  | **Patients who developed hypokalemia before L-AMB treatment initiation** | | |  | **Patients who developed hypokalemia after L-AMB treatment initiation** | | |
| --- | --- | --- | --- | --- | --- | --- | --- |
| **Patient characteristics** | **Adequate K suppl. (N=25)** | **Inadequate K suppl. (N=18)** | **P-value** |  | **Adequate K suppl. (N=23)** | **Inadequate K suppl. (N=15)** | **P-value** |
| Sex, male | 18 (72%) | 9 (50%) | 0.204 |  | 16 (70%) | 11 (73%) | 1.000 |
| Age, years | 66.5±16.5 | 67.4±11.6 | 0.827 |  | **71.4±10.6** | **61.5±15.5** | **0.046** |
| Comorbidities |  |  |  |  |  |  |  |
| Diabetes mellitus | 9 (36%) | 5 (28%) | 0.744 |  | 11 (48%) | 3 (20%) | 0.101 |
| Hypertension | 6 (24%) | 9 (50%) | 0.109 |  | 11 (48%) | 6 (40%) | 0.744 |
| Heart failure | **3 (12%)** | **8 (44%)** | **0.031** |  | 7 (30%) | 2 (13%) | 0.273 |
| Baseline eGFR (mL/min) | 116.6±58.8 | 105.4±54.0 | 0.531 |  | **85.8±28.3** | **113.4±31.0** | **0.012** |
| L-AMB treatment |  |  |  |  |  |  |  |
| Duration (days) | 14.7±16.7 | 18.5±14.1 | 0.433 |  | 33.6±19.0 | 23.3±14.8 | 0.078 |
| Daily dosing (mg/kg/day) | 2.5±0.8 | 2.4±0.6 | 0.701 |  | 2.6±0.5 | 2.6±0.7 | 0.907 |
| Cumulative dosing (mg/kg) | 36.4±38.2 | 48.9±45.2 | 0.362 |  | 88.0±55.2 | 63.0±48.4 | 0.163 |
| Serum K |  |  |  |  |  |  |  |
| Duration of hypokalemia (days) | **2.9±1.9** | **5.8±2.1** | **<0.001** |  | **3.9±4.4** | **13.9±10.0** | **0.003** |
| Minimum K (mEq/L) | 3.1±0.2 | 3.0±0.3 | 0.239 |  | 3.0±0.3 | 3.0±0.3 | 0.582 |
| K level of hypokalemia onset (mEq/L) | 3.1±0.2 | 3.1±0.3 | 0.698 |  | 3.1±0.3 | 3.0±0.3 | 0.446 |
| Average K (mEq/L) | **3.5±0.2** | **3.1±0.3** | **<0.001** |  | **3.7±0.3** | **3.1±0.5** | **<0.001** |
| K suppl. |  |  |  |  |  |  |  |
| Duration of supplementation (days) | 4.1±2.2 | 3.6±2.3 | 0.475 |  | **9.9±9.3** | **3.1±3.7** | **0.004** |
| Duration from hypokalemia onset to K suppl. (days) | 1.4±0.7 | 2.0±1.5 | 0.105 |  | 3.1±4.6 | 2.4±1.0 | 0.504 |
| Daily dosing (mEq/day) | 36.1±20.3 | 42.0±25.2 | 0.428 |  | 41.4±16.1 | 50.2±32.5 | 0.357 |
| Cumulative dosing (mEq) | 151.6±123.3 | 129.5±86.8 | 0.504 |  | **478.3±696.1** | **127.3±149.0** | **0.031** |
| Fungal infection |  |  |  |  |  |  |  |
| Aspergillosis | 9 (36%) | 6 (33%) | 1.000 |  | 8 (35%) | 7 (47%) | 0.514 |
| Candidiasis | 3 (12%) | 2 (11%) | 1.000 |  | 0 (0%) | 1 (7%) | 0.395 |
| Cryptococcosis | 0 (0%) | 0 (0%) | 1.000 |  | 2 (9%) | 0 (0%) | 0.509 |
| Zygomycosis | 0 (0%) | 1 (6%) | 0.419 |  | 0 (0%) | 1 (7%) | 0.395 |
| Neutropenia | 0 (0%) | 0 (0%) | 1.000 |  | 2 (9%) | 1 (7%) | 1.000 |
| Others | 9 (36%) | 6 (33%) | 1.000 |  | 8 (35%) | 2 (13%) | 0.259 |
| Unknown | 4 (16%) | 3 (17%) | 1.000 |  | 3 (13%) | 3 (20%) | 0.663 |
| Treatment department |  |  |  |  |  |  |  |
| Hematology | 17 (68%) | 14 (78%) | 0.731 |  | 21 (91%) | 12 (80%) | 0.365 |
| The internal department except for hematology | 5 (20%) | 4 (22%) | 1.000 |  | 2 (9%) | 3 (20%) | 0.365 |
| The surgical department | 2 (8%) | 0 (0%) | 0.502 |  | 0 (0%) | 0 (0%) | 1.000 |
| Others | 1 (4%) | 0 (0%) | 1.000 |  | 0 (0%) | 0 (0%) | 1.000 |
| Pretreatment potassium-related drugs |  |  |  |  |  |  |  |
| Insulin | 13 (52%) | 8 (44%) | 0.760 |  | 10 (43%) | 2 (13%) | 0.077 |
| ACE inhibitors, ARB | 1 (4%) | 1 (6%) | 1.000 |  | 2 (9%) | 0 (0%) | 0.509 |
| Sodium bicarbonate | 3 (12%) | 3 (17%) | 0.683 |  | 2 (9%) | 0 (0%) | 0.509 |
| Potassium citrate | 0 (0%) | 0 (0%) | 1.000 |  | 0 (0%) | 0 (0%) | 1.000 |
| Diuretic drugs |  |  |  |  |  |  |  |
| Loop | 16 (64%) | 8 (44%) | 0.230 |  | 10 (43%) | 5 (33%) | 0.736 |
| Thiazide | 1 (4%) | 0 (0%) | 1.000 |  | 0 (0%) | 0 (0%) | 1.000 |
| Potassium-sparing | 5 (20%) | 4 (22%) | 1.000 |  | 1 (4%) | 0 (0%) | 1.000 |
| β-agonist | 2 (8%) | 3 (17%) | 0.634 |  | 0 (0%) | 1 (7%) | 0.395 |
| β-blocker | 2 (8%) | 1 (6%) | 1.000 |  | 0 (0%) | 0 (0%) | 1.000 |
| Concomitant potassium-related drugs |  |  |  |  |  |  |  |
| Insulin | 9 (36%) | 6 (33%) | 1.000 |  | 14 (61%) | 4 (27%) | 0.052 |
| ACE inhibitors, ARB | 1 (4%) | 2 (11%) | 0.562 |  | 3 (13%) | 0 (0%) | 0.264 |
| Sodium bicarbonate | 3 (12%) | 2 (11%) | 1.000 |  | 2 (9%) | 0 (0%) | 0.509 |
| Potassium citrate | 0 (0%) | 0 (0%) | 1.000 |  | 0 (0%) | 0 (0%) | 1.000 |
| Diuretic drugs |  |  |  |  |  |  |  |
| Loop | 13 (52%) | 11 (61%) | 0.756 |  | **17 (74%)** | **5 (33%)** | **0.020** |
| Thiazide | 0 (0%) | 0 (0%) | 1.000 |  | 0 (0%) | 0 (0%) | 1.000 |
| Potassium-sparing | 1 (4%) | 4 (22%) | 0.144 |  | **11 (48%)** | **1 (7%)** | **0.012** |
| β-agonist | 0 (0%) | 3 (17%) | 0.066 |  | 1 (4%) | 1 (7%) | 1.000 |
| β-blocker | 2 (8%) | 1 (6%) | 1.000 |  | 0 (0%) | 0 (0%) | 1.000 |

Bold values indicate statistically significant P-values (P<0.05). Adequate potassium supplementation was defined as the correction of serum potassium levels to ≥3.5 mEq/L, while inadequate potassium supplementation was defined as incomplete serum potassium correction (i.e., <3.5 mEq/L). Other fungal infections included unclassified or unspecified mycosis. Categorical variables are presented as frequencies and proportions (%), while continuous variables are expressed as mean ± standard deviation. The Welch’s *t*-test was used to compare two groups for continuous variables, while the Fisher’s exact test was used for two categorical variables. ACE inhibitor/ARB: angiotensin-converting enzyme inhibitor/angiotensin receptor blocker; eGFR: estimated glomerular filtration rate; L-AMB: liposomal-amphotericin B; K: potassium; NA: not analyzed; suppl.: supplementation

**Table S4** Stratified analysis of AKI occurrence in hypokalemic patients adequately supplemented with potassium before L-AMB initiation.

| **Stratification** | | **Patients who developed hypokalemia before L-AMB treatment initiation** | | | | | | | | | | | | |
| --- | --- | --- | --- | --- | --- | --- | --- | --- | --- | --- | --- | --- | --- | --- |
|  |  | **Any stage of AKI (%)** | | |  | **AKI stage 1 (%)** | | |  | **AKI stage 2 or 3 (%)** | | | | |
|  |  | **Adequate K suppl. (N=25)** | **Inadequate K suppl. (N=18)** | **P-value** |  | **Adequate K suppl. (N=25)** | **Inadequate K suppl. (N=18)** | **P-value** |  | **Adequate K suppl. (N=25)** | **Inadequate K suppl. (N=18)** | | **P-value** | |
| Serum K | |  |  |  |  |  |  |  |  |  |  | |  | |
| Minimum K level before K suppl. (mEq/L) | K<3.0 | 4/9 (44%) | 6/8 (75%) | 0.335 |  | 3/9 (33%) | 2/8 (25%) | 1.000 |  | 1/9 (11%) | 4/8 (50%) | 0.131 | |  |
|  | K≥3.0, K<3.5 | 7/16 (44%) | 5/10 (50%) | 1.000 |  | 3/16 (19%) | 3/10 (30%) | 0.644 |  | 4/16 (25%) | 2/10 (20%) | 1.000 | |  |
| K level of hypokalemia onset (mEq/L) | K<3.0 | 4/9 (44%) | 4/6 (67%) | 0.608 |  | 3/9 (33%) | 2/6 (33%) | 1.000 |  | 1/9 (11%) | 2/6 (33%) | 0.525 | |  |
|  | K≥3.0, K<3.5 | 7/16 (44%) | 7/12 (58%) | 0.704 |  | 3/16 (19%) | 3/12 (25%) | 1.000 |  | 4/16 (25%) | 4/12 (33%) | 0.691 | |  |
| K suppl. | |  |  |  |  |  |  |  |  |  |  |  | |  |
| Duration from hypokalemia onset to K suppl. (days) | Day=1 | 8/19 (42%) | 7/10 (70%) | 0.245 |  | 5/19 (26%) | 3/10 (30%) | 1.000 |  | 3/19 (16%) | 4/10 (40%) | 0.193 | |  |
|  | Day>1 | 3/6 (50%) | 4/8 (50%) | 1.000 |  | 1/6 (17%) | 2/8 (25%) | 1.000 |  | 2/6 (33%) | 2/8 (25%) | 1.000 | |  |
| Daily dosing (mEq/day) | Dose≥40 | 6/12 (50%) | 6/10 (60%) | 0.691 |  | 3/12 (25%) | 2/10 (20%) | 1.000 |  | 3/12 (25%) | 4/10 (40%) | 0.652 | |  |
|  | Dose<40 | 5/13 (38%) | 5/8 (63%) | 0.387 |  | 3/13 (23%) | 3/8 (38%) | 0.631 |  | 2/13 (15%) | 2/8 (25%) | 0.618 | |  |
| Pretreatment potassium-related drugs | |  |  |  |  |  |  |  |  |  |  |  | |  |
| Insulin | With | 7/13 (54%) | 6/8 (75%) | 0.400 |  | 4/13 (31%) | 3/8 (38%) | 1.000 |  | 3/13 (23%) | 3/8 (38%) | 0.631 | |  |
|  | Without | 4/12 (33%) | 5/10 (50%) | 0.666 |  | 2/12 (17%) | 2/10 (20%) | 1.000 |  | 2/12 (17%) | 3/10 (30%) | 0.624 | |  |
| Sodium bicarbonate | With | 2/3 (67%) | 2/3 (67%) | 1.000 |  | 0/3 (0%) | 0/3 (0%) | 1.000 |  | 2/3 (67%) | 2/3 (67%) | 1.000 | |  |
|  | Without | 9/22 (41%) | 9/15 (60%) | 0.325 |  | 6/22 (27%) | 5/15 (33%) | 0.728 |  | 3/22 (14%) | 4/15 (27%) | 0.408 | |  |
| ACE inhibitors, ARB | With | 1/1 (100%) | 1/1 (100%) | 1.000 |  | 0/1 (0%) | 1/1 (100%) | 1.000 |  | 1/1 (100%) | 0/1 (0%) | 1.000 | |  |
|  | Without | 10/24 (42%) | 10/17 (59%) | 0.350 |  | 6/24 (25%) | 4/17 (24%) | 1.000 |  | 4/24 (17%) | 6/17 (35%) | 0.270 | |  |
| Loop diuretic drugs | With | 7/16 (44%) | 5/8 (63%) | 0.667 |  | 3/16 (19%) | 3/8 (38%) | 0.362 |  | 4/16 (25%) | 2/8 (25%) | 1.000 | |  |
|  | Without | 4/9 (44%) | 6/10 (60%) | 0.656 |  | 3/9 (33%) | 2/10 (20%) | 0.628 |  | 1/9 (11%) | 4/10 (40%) | 0.303 | |  |
| Potassium-sparing diuretic drugs | With | 4/5 (80%) | 2/4 (50%) | 0.524 |  | 3/5 (60%) | 0/4 (0%) | 0.167 |  | 1/5 (20%) | 2/4 (50%) | 0.524 | |  |
|  | Without | 7/20 (35%) | 9/14 (64%) | 0.163 |  | 3/20 (15%) | 5/14 (36%) | 0.228 |  | 4/20 (20%) | 4/14 (29%) | 0.689 | |  |
| β-agonist | With | 1/2 (50%) | 2/3 (67%) | 1.000 |  | 0/2 (0%) | 1/3 (33%) | 1.000 |  | 1/2 (50%) | 1/3 (33%) | 1.000 | |  |
|  | Without | 10/23 (43%) | 9/15 (60%) | 0.508 |  | 6/23 (26%) | 4/15 (27%) | 1.000 |  | 4/23 (17%) | 5/15 (33%) | 0.436 | |  |
| β-blocker | With | 0/2 (0%) | 1/1 (100%) | 0.333 |  | 0/2 (0%) | 1/1 (100%) | 0.333 |  | 0/2 (0%) | 0/1 (0%) | 1.000 | |  |
|  | Without | 11/23 (48%) | 10/17 (59%) | 0.538 |  | 6/23 (26%) | 4/17 (24%) | 1.000 |  | 5/23 (22%) | 6/17 (35%) | 0.477 | |  |
| Patient background | |  |  |  |  |  |  |  |  |  |  |  | |  |
| Sex | Male | 10/18 (56%) | 5/9 (56%) | 1.000 |  | 5/18 (28%) | 4/9 (44%) | 0.423 |  | 5/18 (28%) | 1/9 (11%) | 0.628 | |  |
|  | Female | 1/7 (14%) | 6/9 (67%) | 0.060 |  | 1/7 (14%) | 1/9 (11%) | 1.000 |  | **0/7 (0%)** | **5/9 (56%)** | **0.034** | |  |
| Age (year) | Year≥65 | 3/14 (21%) | 5/11 (45%) | 0.389 |  | 2/14 (14%) | 3/11 (27%) | 0.623 |  | 1/14 (7%) | 2/11 (18%) | 0.565 | |  |
|  | Year<65 | 8/11 (73%) | 6/7 (86%) | 1.000 |  | 4/11 (36%) | 2/7 (29%) | 1.000 |  | 4/11 (36%) | 4/7 (57%) | 0.630 | |  |
| Baseline eGFR (mL/min) | <60 | 0/1 (0%) | 0/1 (0%) | 1.000 |  | 0/1 (0%) | 0/1 (0%) | 1.000 |  | 0/1 (0%) | 0/1 (0%) | 1.000 | |  |
|  | ≥60 | 11/24 (46%) | 11/17 (65%) | 0.342 |  | 6/24 (25%) | 5/17 (29%) | 1.000 |  | 5/24 (21%) | 6/17 (35%) | 0.476 | |  |
| Diabetes mellitus | With | 4/9 (44%) | 4/5 (80%) | 0.301 |  | 2/9 (22%) | 3/5 (60%) | 0.266 |  | 2/9 (22%) | 1/5 (20%) | 1.000 | |  |
|  | Without | 7/16 (44%) | 7/13 (54%) | 0.715 |  | 4/16 (25%) | 2/13 (15%) | 0.663 |  | 3/16 (19%) | 5/13 (38%) | 0.406 | |  |
| Hypertension | With | 3/6 (50%) | 8/9 (89%) | 0.235 |  | 1/6 (17%) | 4/9 (44%) | 0.580 |  | 2/6 (33%) | 4/9 (44%) | 1.000 | |  |
|  | Without | 8/19 (42%) | 3/9 (33%) | 1.000 |  | 5/19 (26%) | 1/9 (11%) | 0.630 |  | 3/19 (16%) | 2/9 (22%) | 1.000 | |  |
| Heart failure | With | 1/3 (33%) | 6/8 (75%) | 0.491 |  | 1/3 (33%) | 2/8 (25%) | 1.000 |  | 0/3 (0%) | 4/8 (50%) | 0.236 | |  |
|  | Without | 10/22 (45%) | 5/10 (50%) | 1.000 |  | 5/22 (23%) | 3/10 (30%) | 0.681 |  | 5/22 (23%) | 2/10 (20%) | 1.000 | |  |

Bold values indicate statistically significant P-values (P<0.05). Adequate potassium supplementation was defined as the correction of serum potassium levels to ≥3.5 mEq/L, while inadequate potassium supplementation was defined as incomplete serum potassium correction (i.e., <3.5 mEq/L). Variables are expressed as frequencies and proportions (%). Total patient number is presented as the denominator. P-values were calculated using the Fisher’s exact test. ACE inhibitor/ARB: angiotensin-converting enzyme inhibitor/angiotensin receptor blocker; eGFR: estimated glomerular filtration rate; L-AMB: liposomal-amphotericin B; K: potassium; suppl.: supplementation
